# Forensic aspects of trauma to the male external genitalia (TMEG), analysis of a series of 84 cases

**DOI:** 10.1016/j.amsu.2022.103916

**Published:** 2022-06-06

**Authors:** A. Kbirou, I. Jandou, M. Sayah, H. Benhadda, A. Moataz, M. Dakir, A. Debbagh, R. Aboutaieb

**Affiliations:** a Urology Department, Ibn Rochd University Hospital Center, Casablanca, Morocco; b Faculty of Medicine and Pharmacy of Casablanca, Hassan II University, Casablanca, Morocco; c Sexual and Reproductive Health Laboratory, Faculty of Medicine and Pharmacy, Hassan II University, Casablanca, Morroco

**Keywords:** Trauma, Penis, Bursa, Forensic aspects

## Abstract

**Objectives:**

Describe the medico-legal aspects of trauma to the male external genitalia by specifying their epidemiological, clinical, paraclinical, therapeutic and evolutionary characteristics.

**Materials and methods:**

Retrospective study spread over a period of 3 years (2017–2019) concerning adult patients with trauma to the male external genitalia consulting in the surgical emergency and forensic medicine departments of the Ibn Rochd University Hospital Center in Casablanca.

**Results:**

We collected 84 cases of TMEG within the framework of evaluation of bodily injury aimed at fixing the duration of Total Temporary Incapacity (TTI) and the rate of Permanent Partial Incapacity (PPI). There was a predominance of bursa trauma (85.7%) followed by penis trauma (14.5%). The average age of the patients was 39 years (17–61 years). Road accidents are the cause of 42% of cases. Regarding bursa trauma, the clinical symptomatology was dominated by pain (100%) and increased scrotal volume (50%). Scrotal ultrasound found the main lesion to be scrotal hematomas (35.71%) followed by ruptures of the tunica albuginea (9.5%). Surgical exploration was indicated in 45.23% of cases, of which (38%) was conservative treatment.

For trauma to the penis, the clinical examination revealed 1 case of fracture of the cavernous body, a hematoma of the penis in only 1 patient, a superficial wound of the penis in 1 patient and the rest of the patients (9 cases) showed no lesions. Surgical treatment was indicated in only one patient. The average duration of temporary total incapacity was 17.5 days and the average rate of partial permanent incapacity was 4%.

**Conclusion:**

Physicians who are experts in the evaluation of bodily injury are frequently confronted with the evaluation of the damage resulting from the TMEG. The medico-legal evaluation of these traumas remains difficult and very varied requiring a perfect knowledge of the mechanisms of these traumas, of the therapeutic and evolutionary principles helping in the judicial decision.

## Highlights

• Trauma to the male external genitalia is infrequent and mainly affects a young population.
• Early diagnostic and therapeutic management determines the functional results.
• These complications are serious and frequent involving the sexuality and fertility of patients.
• Early surgical treatment has clearly improved the prognosis of this type of trauma.
• The medico-legal evaluation of this type of trauma is difficult requiring a multidisciplinary approach.

## 1 Introduction

Urogenital trauma has an incidence of between 10 and 20% [1,2]. In terms of frequency, these traumas rank third among traumatic pathology in urology, after kidney and bladder trauma, at a level close to urethral trauma [3,4]. Trauma to the scrotum represents two-thirds of TMEG and trauma to the penis one-third [3,4]. They constitute one of the frequent etiologies of scrotal pain in the emergency room. The circumstances of occurrence are multiple represented by assaults, road accidents, accidents at work, sports and carelessness. The paraclinical examinations allow better management of the TMEG without forgetting their diagnostic value as much as the documents comprising the lesion assessment imputed to the trauma and their medico-legal interest affirming the seriousness of the lesions and the damages corresponding thereto. Early surgical treatment has considerably improved the prognosis of these traumas and reduced the rate of complications threatening the fertility and sexuality of these patients [3]. In the long term, these complications make difficult, in certain situations, the medico-legal evaluation of this type of trauma requiring consultation between the attending physician and the medical assessor or pathologist. There is little data concerning this type of trauma not only in the literature but also in our African context.

The aim of our work was to analyze the medico-legal evaluation of the TMEG by taking into account the clinical signs, the lesion assessment, the therapeutic choice and the damages in the short (TTI) to and in the long term (PPI) which have been granted to them. This work has been reported according to SCARE 2020 criteria [5].

## 2 Materials and methods

This is a descriptive retrospective study spread over 3 years, from January 1, 2017 to December 31, 2019, including 84 adult patients with TMEG consulting in the urology department and the medico-judicial unit of Ibn Rochd Hospital center of Casablanca.

The participants described the mechanisms, the clinical signs, the ultrasound data, the treatment methods indicated, the progressive signs and the medico-legal prejudices (Table 1). This study registered at http://www.researchregistry.com. Research Registry UIN: researchregistry 7783.

**Table 1:** Description of the different variables studied.

Epidemiological study	Type of injury, number of cases, age,
Clinical study	Clinical signs, clinical examination
Radiological study	Ultrasound Results
Therapeutic and evolutionary study	Therapeutic modalities, indications and complications
Forensic assessment	Total temporary incapacity, Partial permanent incapacity

The anamnesis specified data concerning the age, the mechanism of the trauma as well as these circumstances, then the clinical examination was carried out, highlighting the clinical signs orienting the paraclinical study and the therapeutic attitude. The treatment was dual medical and surgical. Before each operative gesture, we insisted on providing complete information to the patients on the various operative risks, especially concerning fertility and sexuality, and taking their concentration. The surgery was performed in the surgical emergency room without any particular anesthetic instructions. Whatever the therapeutic choice, regular monitoring is required to objectivise the complications.

After the acute phase, the patients consulted with the medical examiners for forensic evaluation of their traumas, specifying the duration of total temporary incapacity and the rate of partial permanent incapacity.

Regarding the research methodology used, a quantitative analysis of the data collected from medical records and forensic certificates was carried out, respecting the anonymity of the patients and taking their consent.

The comparison of the studied variables was made by the Chi.2 test, the differences of which were considered significant for p < 0.05.

In order to conduct this study, we received the agreement of the ethics committee of the Ibn Rochd hospital in accordance with the recommendations of the faculty of medicine of the university hassan II of casablanca.

## 3 Results

**Fig. 1:**
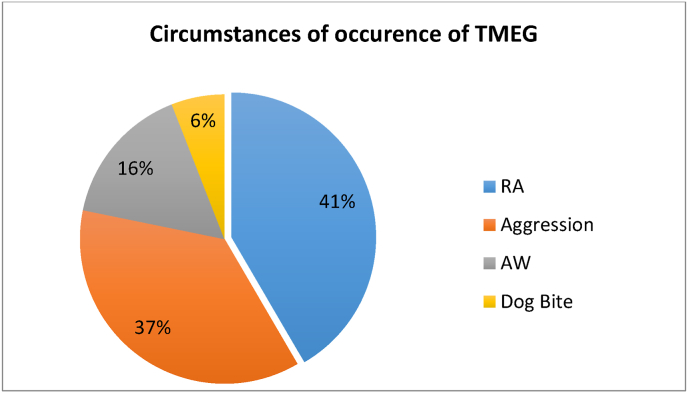
Distribution of patients according to the circumstances of occurrence of TGEM.

We collected 84 cases whose average age was 39 years with extremes of 17 and 61 years. The main circumstances of occurrence were Road Accidents (RA) in 35 cases (42%) (p < 0.003), followed by assaults in 31 cases (37%) (p < 0.006), accidents at work (AW) in 13 cases (16%) (p < 0.001) and dog bites in 5 cases (6%) (p < 0.004)(Fig. 1). There was a predominance of scrotal trauma in 72 cases (85.71%) (p < 0.031) while 12 cases (14.28%) (p < 0.042) had presented trauma to the penis. Concerning bursa trauma (BT), it is essentially a blunt trauma in 61 cases (72.61%) (p < 0.053) and open trauma in 11 cases (13%) (p < 0.073). While it was isolated in the majority of cases (66 cases° (78.57%) (p < 0.046) and associated with other trauma in 7 cases (8%) (p < 0.071).

**Fig. 2:**
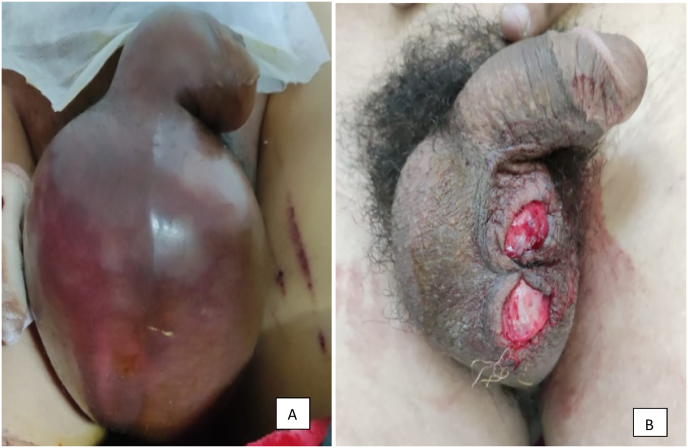
Aspect in favor of bursa trauma A- Post-traumatic testicular hematoma secondary to a public road accident B- Open trauma of the bursae caused by a dog bite.

Clinically, all patients presented with pain and 42 patients (50%) (p < 0.065) had an enlarged scrotum and 21 patients (25%) (p < 0.034) had a hematocele (Fig. 2). Ultrasound found the main abnormalities of scrotal hematoma in 30 patients (35.71%) (p < 0.023) and intra-testicular hematomas and hematoceles in 16 cases (19%) (p < 0.013) and ruptures of tunica albuginea in 8 patients (9.5%) (p < 0.080)(Table 2). All patients received medical treatment with analgesics and antibiotics. Surgical treatment was indicated in 38 patients with recourse to conservative treatment in 36 patients (43%) (p < 0.054) which consisted of evacuation of scrotal and testicular hematomas in 32 patients (38%) (p < 0.044) and 4 patients (4.71%) (p < 0, 060) benefited from a pulp resection with suturing of the tunica albuginea (Fig. 2). Orchiectomy was performed in 2 patients (2.3% of cases) (p < 0.050). The postoperative follow-up was simple. The evolution was marked by the occurrence of testicular atrophy in two patients (2.7%) (p < 0.034). The average duration of temporary total disability was 27.5 days with extremes of 5 and 60 days, while the average permanent partial disability was 2.5% (p < 0.001) with extremes of 0 and 5% (p < 0.003).

**Table 2:** Distribution of patients according to clinical and ultrasound signs of BD.

	signs	Number of cases	%	p
Clinical signs	Scrotal pain	72	100	0.097
	big purse	42	50	0.065
	Hematocele.	21	25	0.034
Ultrasound signs	Scrotal hematoma	30	35.71	0.023
	Intratesticular hematomas and hematoceles	16	19	0.013
	Tunica albuginea ruptures	8	9.5	0.080

Concerning penile trauma, the injury assessment showed 1 case of cavernous body fracture (2%) (p < 0.0071), hematoma of the penis in 1 patient (2%) (p < 0.0021), a superficial wound of the penis and the rest of the patients (9 cases)(10,7%) (p < 0.0074) did not present lesions. Surgical treatment was indicated in only 1 patient (2%) (p < 0.008) and consisted of suturing the tunica albuginea. The evolution was favorable in all the patients. The average duration of temporary total disability was 17.5 days with extremes of 5 and 30 days, while the average permanent partial disability was 4% (p < 0.008) with extremes of 0 and 8%. The average duration of consolidation varies according to the lesion assessment. There were 20 days as the average duration for the hematocele and 32,5 days for fracture of the penis,22,5 days for Hematoma of the penis and 35 days for Wound of the penis (Table 3).

**Table 3:** Distribution of the duration of consolidation according to the type of lesions.

Type of lesion	Duration of consolidation	P
Hematocele	15–25 days	P < 0.087
tunica albuginea rupture	25–45 days	P < 0.098
Cavernous body fracture	25–50 days	P < 0.076
Hematoma of the penis	15–30 days	P < 0.054
Wound of the penis	25–45 days	P < 0.032

## 4 Discussion

Relatively rare, the incidence of TGEM is probably underestimated [7]. We do not know, in fact, the number of patients with minor lesions who are medically treated by emergency services and general practitioners, or who do not consult, and who therefore do not appear in the series of patients taken into workload in urology departments [7]. The age of predilection for this type of trauma is between 20 and 30 years [5]. The average age in our study was 39 years higher than that reported by the literature which was 25 and 28 years respectively [5,6]. The main causes are public road accidents (bicycles, mopeds and motorcycles in more than 80% of cases), assaults (kicks at the point of scrotal impact), work accidents (falls, crushing, machine accidents) and sports accidents (ball sport, combat sport) [[3], [4], [5],^8^,^9^]. However, we must not forget the particular case of paraplegic patients who constitute a population at risk of traumatic lesions from GEM, especially during their daily care [6].

Concerning the BT, the lesion assessment, based on the clinical examination and completed by the scrotal ultrasound which constitutes an essential paraclinical examination especially in the event of diagnostic doubt, objectified lesions of the scrotum in the type of ecchymosis and scrotal hematoma. Other testicular lesions may occur such as testicular contusion, which represents the most common lesion (85%) [8], intra-testicular hematoma, testicular dislocations and testicular fractures (rupture of the tunica albuginea) [ 2]. In our study, we found that the scrotal hematoma represented the main lesion (35%). This observation is similar to the series of Benchekroun and his collaborators [10]. Surgical treatment is indicated in the presence of a hematocele, testicular hematoma or testicular fracture [11]. Furthermore, monitoring is indicated in the absence of large bursae, clinical hematocele and testicular integrity on ultrasound [12]. Follow-up enables the detection of complications, including frequently reported testicular atrophy (50%), fertility disorders (75%) and infectious complications [14,15]. Concerning the Penis Traumas (PT), we can distinguish three types of traumas which differ by their etiology, the lesion assessment and their treatment. First, the fracture of the penis occurring as a result of forced manipulations during erection (masturbation, camouflage of an erection, missteps or accident of coitus). Their diagnosis is clinically evoked in front of the swollen aubergine aspect of the penis. Treatment is mainly surgical by suturing the defect of the tunica albuginea [13].

In the 2nd place is also seen the strangulations which can evolve from the cutaneous ulceration to the urethral fistula. Choking agents are varied (string, rubber band or hair) [18].

Finally, rare but serious amputations requiring psychiatric evaluation. Early reimplantation under microsurgery is possible with good aesthetic and functional results [18,19].

However, other lesions may exist such as wounds and lacerations of the sheath of the penis caused by domestic and sports accidents [18,19].

In our study, we collected 1 case of cavernous body fracture and 1 hematoma of the penis while Diabaté reported on a series of 15 PTs with open trauma (wounds, lacerations and amputations) as the main clinical form (73%) [18].

The medico-legal aspects of TGEM have been discussed because of their personal and family repercussions. The forensic pathologist must first establish the causal link between the initial trauma and the traumatic lesions by specifying the mechanism of the trauma which can sometimes be difficult in our Moroccan socio-cultural context. He must also report the precise lesional assessment based on the clinical and paraclinical examinations and the treatment undertaken, which enabled him to write the initial medical certificate specifying the incapacity for Personal Work (ITW). This entity should be assessed carefully and objectively due to the legal implications. The ITW quantifies the impact of the trauma and allows the magistrate to qualify the offense [16]. In our study, the average duration of the ITW is 27.5 days. This duration is qualified according to the intention of the author of the trauma. In the case of intentional blows and injuries, the ITW is limited to 20 days, 6 days in the case of unintentional blows and injuries and 30 days in the case of RA [16]. Referral to the attending physician is essential to determine the date of consolidation when the lesions take on a permanent character and when treatment is no longer necessary, except to avoid aggravation, and what makes it possible to assess the degree permanent functional incapacity resulting in non-patrimonial damage. The damage linked to the TGEM is twofold: sexual function resulting in impaired sexual activity and reproductive function linked to the difficulty or even the impossibility of having children [20,21]. Unfortunately sexual harm continues to have an exceptional character in Morocco because of the socio-cultural characteristics of our society where sexuality and fertility constitute a taboo subject, which pushes the victims to hide this harm and without forgetting the ambiguity of the legislative texts dealing with these damages. However, legal compensation for specifically sexual harm is more and more frequently pleaded before the courts, but its concept is not entirely clarified. Unfortunately sexual harm continues to have an exceptional character in Morocco because of the socio-cultural characteristics of our society where sexuality and fertility constitute a taboo subject, which pushes the victims to hide this harm and without forgetting the ambiguity of the legislative texts dealing with these damages. However, legal compensation for specifically sexual harm is more and more frequently pleaded before the courts, but its concept is not entirely clarified. Unfortunately sexual harm continues to have an exceptional character in Morocco because of the socio-cultural characteristics of our society where sexuality and fertility constitute a taboo subject, which pushes the victims to hide this harm and without forgetting the ambiguity of the legislative texts dealing with these damages. However, legal reparation for specifically sexual harm is more and more frequently pleaded before the courts, but its concept is not entirely clarified.

Concerning the final certificate, the medical examiner after the opinion of the attending urologist must determine the permanent partial incapacity which represents an instrument for measuring the bodily injury by using an indicative scale and constitutes an indicator of proof of the damages invoked apprehended on a descriptive mixed mode (description of sequelae and their repercussions on daily and professional life) and mathematical mode, representing the percentage of loss of activity based on the functional scale of disabilities [16,17]. For severe BT, the PPI rate is 5% in the event of loss of a single testicle, whereas in the event of bilateral castration or sterility the rate is 30% [16,[17], [24]].

However, the PPI is between 50 and 60% for serious trauma to the penis (loss of the penis) and between 60 and 80% in the event of emasculation (loss of the testicles and penis) according to French legislation [22,[23], [24]].

The main limitations of this study were represented by the unrepresentative sample given the limited number of patients included in this study and the limited data in the literature concerning scientific publications on this subject. While this work is a challenge not only for our team but all researchers, regardless of urologist and/or medical examiner, we encourage scientific research on the medical evaluation of this type of trauma.

## 5 Conclusion

The medico-legal evaluation of GE trauma is difficult due to the diversity of mechanisms and elementary lesions found in the lesion assessment suspected by clinical and radiological examination and confirmed by surgical exploration, making coordination between the various stakeholders essential (forensic doctor, urologist and radiologist). This work underlines the interest of drafting a scale of total temporary incapacities that do not exist in Morocco.

## Ethical approval

The study committee of the chu ibn Rochd hospital center approves the favorable opinion to publish this work.

## Sources of funding

This study did not receive any sources of funding

## Author contribution

Dr. AK, Dr. MS, and Dr. IJ analysed and performed the literature research,Pr.AM, Pr. MD, Pr.AD and RA performed the examination and performed the scientific validation of the manuscript. Issam Jandou was the major contributors to the writing of the manuscript. All authors read and approved the manuscript.

## Trail registry number

Name of the registry: researchregistry.

Unique identifying number or registration ID: 7783.

Hyperlink to your specific registration (must be publicly accessible and will be checked):

## Guarantor

Dr KBIROU Adil.

## Declaration of competing interest

All authors disclose any conflicts of interest.

## Provenance and peer review

Not commissioned, externally peer-reviewed.

## Patient confidentiality

The references, figures, tables and any acknowledgements include not any confidential identifying patient information, such as the name or date of birth of the patient.

## Consent

Written informed consent was obtained from the patient for publication of this case series and accompanying images. A copy of the written consent is available for review by the Editor-in-Chief of this journal on request.

## Declaration of competing interest

No Conflicts of interest.

## Abbreviations list

**TMEG**: Trauma to the Male External Genitalia ()
**PPI**: Permanent Partial Incapacity
**TTI**: Total Temporary Incapacity
**AW**: Accidents at Work
**RA**: Road Accidents
**BT**: Bursa Trauma
**PT**: Penis Trauma
**ITW**: Incapacity for Personal Work
